# Evaluation of the ResistancePlus MG FleXible Assay for Detection of Wild-Type and 23S rRNA-Mutated Mycoplasma genitalium Strains

**DOI:** 10.1128/JCM.01900-19

**Published:** 2020-02-24

**Authors:** Suhella Tulsiani Drud, Peter Njuguna, Samantha Ebeyan, Simon Erskine, Mette Holm, Susanne Cramer Johansson, Lit Yeen Tan, Jorgen Skov Jensen

**Affiliations:** a Department of Bacteria, Parasites and Fungi, Statens Serum Institut, Copenhagen, Denmark; b SpeeDx Pty. Ltd., National Innovation Centre, Eveleigh, NSW, Australia; Marquette University

**Keywords:** *Mycoplasma genitalium*, detection, macrolide resistance, GeneXpert Dx, ResistancePlus MG FleXible, diagnostics, molecular assay, near-patient

## Abstract

Antibiotic resistance in Mycoplasma genitalium is rising globally, and resistance-guided diagnostics can facilitate targeted and timely treatment. The ResistancePlus MG FleXible (RPMG Flex) assay for the detection of M. genitalium and macrolide resistance-mediating mutations (MRMM) was evaluated for analytical sensitivity, specificity, reproducibility, and inhibition in the presence of interfering substances by simulating M. genitalium-negative pooled urine and swab matrices with M. genitalium cultures. Furthermore, the clinical sensitivity of the assay was evaluated and compared with a reference real-time PCR assay. The analytical sensitivity of the RPMG Flex assay was 157 genomes/ml for wild-type (WT) and 387 genomes/ml for MRMM strains in both matrices. For clinical specimens, the RPMG assay had an overall sensitivity of 96.1% (95% urine: 10/10 WT, 9/10 MRMM; 96.5% swab: 25/26 WT, 26/29 MRMM) compared to 85.7% for the MgPa/MagNAPure24 assay (95% urine: 19/20; 87% swab: 48/57). Clinical specificity was 100% for urine and 98.5% for swab specimens, respectively. No inhibition due to the presence of any of the tested interfering substances was observed. The RPMG Flex assay was more sensitive than the reference MgPa assay, in particular, for swab specimens. The implementation of this assay may increase ease of use and considerably decrease hands-on time for sample preparation compared to a standard block-based assay. The RPMG Flex assay for the GeneXpert Dx system provides a much-needed platform for the simultaneous detection of MG and MRMM and may thereby facilitate resistance-guided therapy for M. genitalium infections.

## Abstract (precis)

Antibiotic resistance in Mycoplasma genitalium is rising globally, and resistance-guided diagnostics can facilitate targeted and timely treatment. The ResistancePlus MG FleXible (RPMG Flex) assay for the detection of M. genitalium and macrolide resistance-mediating mutations (MRMM) was evaluated for analytical sensitivity, specificity, reproducibility, and inhibition in the presence of interfering substances by simulating M. genitalium-negative pooled urine and swab matrices with M. genitalium cultures.

## INTRODUCTION

Mycoplasma genitalium is a common sexually transmitted bacterium which leads to symptomatic or asymptomatic infections in males and females (1). In males, urethritis and in females, cervicitis and pelvic inflammatory disease, are the most common manifestations that have been associated with M. genitalium (1, 2). Historically, M. genitalium infections were easily treated with the macrolide class of antibiotics; however, recent years have seen an increase in the prevalence of macrolide-resistant M. genitalium infections worldwide (3). In such cases, treatment involves the use of fluoroquinolones such as moxifloxacin as second-line antibiotics (4), although resistance for these has also increased (5).

As with most treatable bacterial infections, rapid and accurate diagnosis of M. genitalium combined with targeted treatment are pivotal to a successful outcome. Diagnosis of M. genitalium infection is reliant on nucleic acid amplification tests because the organism is extremely difficult to culture and because reliable serology has not been developed for diagnostic use. According to most clinical guidelines, detection of M. genitalium should be followed by subsequent detection of macrolide resistance-mediating mutations (MRMM) of the 23S rRNA gene (4). This is commonly achieved either via a second PCR and sequencing (6, 7), via detection by various probe assays (8), or by melt curve analysis of amplicons (9). Diagnosing patients with macrolide-resistant M. genitalium via multiple PCR tests can be both time- and cost-inefficient. A primary diagnostic assay for M. genitalium and its macrolide-resistance status that is both rapid and sensitive will be invaluable from a public health perspective (10).

The rapid, point-of-care GeneXpert (Cepheid, Sunnyvale, CA, USA) instrument system integrates sample purification, nucleic acid amplification, real-time PCR detection, and reporting of results. The system consists of an instrument and a personal computer with preloaded software to run assays and view results. The system has been in use for the detection of a range of pathogens, including methicillin-resistant Staphylococcus aureus (MRSA) (11), Mycobacterium tuberculosis complex with rifampin resistance screening (12), and more recently, chlamydia and gonorrhea (13).

A multiplex PCR assay (ResistancePlus MG; SpeeDx Pty. Ltd., NSW, Australia) for the simultaneous detection of M. genitalium and five MRMM has shown significant potential (14). This assay has recently been adapted for use on the GeneXpert Dx system and is CE-marked and sold as the ResistancePlus MG FleXible assay (hereinafter referred to as RPMG Flex). In the present study, we provide findings from an early evaluation of the RPMG Flex assay for the detection of M. genitalium and MRMM in clinical and simulated samples. Furthermore, we provide data on head-to-head comparison with a conventional block-based real-time PCR assay.

The availability of a resistance-guided assay suitable for near-patient testing will facilitate targeted and timely antibiotic therapy for patients and limit unnecessary treatment with moxifloxacin, which has received warning labels for toxicity, limiting its use to infections where other treatment options are unavailable.

## MATERIALS AND METHODS

### ResistancePlus MG Flexible assay.

The RPMG Flex assay (not available for sale in the United States) for the GeneXpert (SpeeDx Pty. Ltd., Australia) is a real-time PCR assay employing PlexZyme and PlexPrime technology (14) to enable high-level multiplexing of targets, including antimicrobial resistance markers. The assay detects the following three targets: (i) M. genitalium through detection of the M. genitalium adhesion (MgPa) gene, (ii) four mutations in the 23S rRNA gene of M. genitalium (A2058G, A2059G, A2058T, and A2058C; Escherichia coli numbering) that cause resistance to azithromycin, and (iii) an internal control to monitor extraction efficiency and quantitative PCR (qPCR) inhibition. The RPMG Flex assay evaluated in this study utilizes single-use FleXible cartridges which are loaded with PCR mastermix prior to use. This differs from Xpert *in vitro* diagnostics (IVD) tests (Cepheid), where only the sample needs to be loaded into the cartridge. The user adds the MG FleXible reaction mix (44 μl) to the reagent chamber and 1 ml of sample and 10 μl of internal control cells to the sample chamber. The FleXible cartridge is then loaded on the GeneXpert Dx instrument, where both extraction and amplification are performed automatically. An assay definition file (ADF) containing parameters for extraction, PCR cycling conditions, probe check, and result interpretation algorithms is also supplied by the manufacturer. The interpretation of results from the assay is automated by the GeneXpert Dx system software from measured fluorescent signals and embedded calculation algorithms to determine the detection of M. genitalium and 23S rRNA mutations.

### Reference material.

Strains representing each of the M. genitalium 23S rRNA mutation types were cultured as previously described (15). The strains used were G37 (wild-type [WT]), M6302 (A2058C), M6593 (A2059G), M6604 (A2058G), and M6926 (A2058T). M. genitalium cultures were diluted in negative clinical specimen matrix to simulate positive urine and vaginal swab samples. M. genitalium cultures in each matrix were quantitated using the M. genitalium qPCR as previously described (16). Quantity was expressed as genomes/ml as determined from a standard curve generated by dilution of purified M. genitalium G37 DNA.

### Analytical sensitivity and inclusivity testing.

The analytical sensitivity of the RPMG Flex kit was determined by testing dilutions of each representative M. genitalium strain in urine. Dilutions were run over multiple days using 2 kit lots. Probit analysis was performed to determine the lowest dilution that could be detected for each representative M. genitalium strain (limit of detection [LOD]) with at least 95% probability. The LOD established in urine for each representative M. genitalium strain was confirmed in swab matrix by detecting each strain with at least 95% probability from a total of 20 replicates. In addition, dilutions of M. genitalium WT strain (G37) in urine and swab matrices were simultaneously tested with the block-based MgPa PCR assay to provide parallel data on LOD for the easyMAG and MagNAPure systems.

For inclusivity testing, eight additional M. genitalium strains representing M. genitalium 23S rRNA mutation types, as well as geographical and temporal diversity, were tested at 3 × LOD (Table 1). Each strain was diluted into a matrix of negative urine and swab specimens.

**TABLE 1:** M. genitalium strains tested as part of inclusivity^*a*^

M. genitalium 23S rRNA mutation type	Strain	Sample yr	Geographical location
WT	M2300	1991	Denmark
WT	M2321	1991	Denmark
WT	M2341	1991	Denmark
WT	M30 Early	1980	United Kingdom
A2058G	M6271	2004	Australia
A2059G	M6303	2003	Norway
A2059G	M6320	2004	Australia
A2058C	M6848	2008	USA

a All strains were tested at 2 × LOD.

Cross-reactivity of the RPMG Flex kit with M. genitalium containing the A2059C 23S rRNA mutation type (not available as a cultured strain) was evaluated by using a synthetic DNA construct containing M. genitalium MgPa and A2059C 23S rRNA targets. The synthetic target was tested at 5,000 copies per sample in a background of 35 ng human genomic DNA (Promega).

### Analytical specificity.

A total of 42 nontarget microorganisms commonly found in the urogenital system or closely related to M. genitalium were assessed (in triplicate) for potential cross-reactivity with the RPMG Flex assay. Microorganisms were tested at high concentrations as shown in Table 2.

**TABLE 2:** Phylogenetically related and nontarget organisms tested for analytical specificity^*a*^*^,^*^*b*^

Organism	Assay result
Actinomyces israelii	ND
Bacteroides fragilis	ND
Bifidobacterium adolescentis	ND
Campylobacter jejuni	ND
Candida albicans^*c*^	ND
Candida glabrata	ND
Candida parapsilosis	ND
Candida tropicalis^*c*^	ND
Chlamydia trachomatis	ND
Clostridium perfringens	ND
Corynebacterium genitalium	ND
Enterobacter aerogenes	ND
Enterobacter cloacae	ND
Enterococcus faecalis	ND
Fusobacterium nucleatum	ND
Haemophilus ducreyi	ND
Herpes simplex virus I	ND
Herpes simplex virus II	ND
Human papillomavirus type 18 (HeLa cells)^*d*^	ND
Klebsiella oxytoca	ND
Lactobacillus acidophilus	ND
Lactobacillus crispatus	ND
Lactobacillus jensenii	ND
Lactobacillus vaginalis	ND
Listeria monocytogenes	ND
Mycobacterium smegmatis	ND
Mycoplasma hominis	ND
Mycoplasma pneumoniae	ND
Neisseria gonorrhoeae	ND
Pentatrichomonas hominis^*e*^	ND
Peptostreptococcus anaerobius	ND
Prevotella bivia	ND
Propionibacterium acnes^*c*^	ND
Proteus mirabilis	ND
Proteus vulgaris	ND
Pseudomonas aeruginosa	ND
Staphylococcus aureus	ND
Staphylococcus saprophyticus	ND
Streptococcus agalactiae	ND
Streptococcus pyogenes	ND
Trichomonas vaginalis^*e*^	ND
Ureaplasma urealyticum^*c*^	ND

a All organisms were tested at 1 × 10^6^ genomes/ml except where indicated.

b ND, M. genitalium not detected; 23S rRNA mutation not detected.

c Tested at 1 × 10^5^ genomes/ml.

d Quantified as PFU/ml.

e Quantified as CFU/ml.

### Interfering substances.

The performance of the RPMG Flex assay was assessed in the presence of substances or conditions that may be present in urine or swab specimens (Table 3). All substances were tested in the absence of M. genitalium and in the presence of a representative M. genitalium mutant strain (A2058G mutant; M6604 strain) at 3 × LOD. A substance was considered noninterfering when all replicates containing the substance were correctly identified at the concentrations tested. Where interference was observed, testing was repeated with samples containing a lower test amount of the substance until interference was not observed.

**TABLE 3:** Substances tested to assess interference in the performance of the ResistancePlus MG FleXible assay

Sample matrix	Class/substance	Product name	Test concn	Assay result for:^*a*^	
				M. genitalium-negative samples	M. genitalium-positive samples (M6604 strain)
Urine	Whole blood	NA^*e*^	0.4% (vol/vol)^*b*^	ND	D
Urine	Semen	NA	5.0% (vol/vol)	ND	D
Urine	Mucus	Mucin	0.8% (wt/vol)	ND	D
Urine	Antibiotic	Azithromycin	1.8 mg/ml	ND	D
Urine	Analgesic	Paracetamol	3.2 mg/ml	ND	D
Urine	Intravaginal hormones	Progesterone; estradiol	7 mg/ml Progesterone + 0.07 mg/ml beta estradiol	ND	D
Urine	Leukocytes	NA	10^5^ cells/ml	ND	D
Urine	Albumin	Bovine serum albumin	10 mg/ml	ND	D
Urine	Glucose	NA	10 mg/ml	ND	D
Urine	Bilirubin	NA	0.18 mg/ml^*c*^	ND	D
Urine	Acidic urine (pH 4.0)	Urine + *N*-acetyl-l-cysteine	pH 4.0	ND	D
Urine	Alkaline urine (pH 9.0)	Urine + ammonium citrate	pH 9.0	ND	D
Swab	Over-the-counter vaginal products and contraceptives	Vagisil anti-itch crème (1.0 oz)	0.25% (wt/vol)	ND	D
Swab	Over-the-counter vaginal products and contraceptives	K-Y Jelly (4.0 oz)	0.25% (wt/vol)	ND	D
Swab	Over-the-counter vaginal products and contraceptives	Options Gynol II vaginal contraceptive gel	0.25% (wt/vol)	ND	D
Swab	Over-the-counter vaginal products and contraceptives	Walgreens clotrimazole vaginal cream (1.5 oz)	0.25% (wt/vol)	ND	D
Swab	Over-the-counter vaginal products and contraceptives	Vagi-gard douche	0.25% (wt/vol)	ND	D
Swab	Over-the-counter vaginal products and contraceptives	Vagisil ProHydrate natural feel internal moisturizing gel (0.2-oz 8 pack)	0.25% (wt/vol)	ND	D
Swab	Over-the-counter vaginal products and contraceptives	Vagisil daily intimate deodorant powder (8.0 oz)	0.10% (wt/vol)^*d*^	ND	D
Swab	Deodorant and powders	Summer's Eve deodorant spray (2.0 oz)	0.25% (vol/vol)	ND	D
Swab	Hemorrhoidal cream	Preparation H hemorrhoidal cream (0.9 oz)	0.25% (wt/vol)	ND	D
Swab	Prescription-only medicines	Estrace (Estradiol vaginal cream, USP 0.01%)	0.25% (wt/vol)	ND	D

a D, output result “M. genitalium detected; 23S rRNA mutation detected”; ND, output result “M. genitalium not detected; 23S rRNA mutation not detected.”

b Interference may be observed in samples containing >0.4% whole blood.

c Interference may be observed in samples containing >0.18 mg/ml bilirubin.

d Interference may be observed in samples containing >0.1% wt/vol Vagisil intimate powder.

e NA, not applicable.

### Reproducibility.

The reproducibility of the RPMG Flex assay was assessed across reagent lots, runs, operators, and days using identical panels consisting of urine and vaginal swab samples. Three lots of the RPMG Flex assay were included in the study.

### Clinical specimens.

The sample collection consisted of 177 deidentified specimens from male and female patients, collected for M. genitalium diagnostic testing in Denmark and Sweden from 2009 to 2015, which had been stored at –20°C since the original diagnostic testing was completed. This included 40 first-void urine samples (32 male and 8 female) and 137 genital swab samples (69 cervical, 35 vaginal, and 33 urethral [11 female, 22 male]). All samples had previously been characterized with a laboratory-developed test (LDT; MgPa PCR) (16) to determine the M. genitalium status and a PyroMark sequencing LDT (7) to determine 23S rRNA status (reference testing result). To enable a sufficient volume for simultaneous testing with RPMG Flex and the two repeat DNA extractions for the block-based MgPa assay, swab specimens were diluted with Copan universal transport medium (UTM; Copan Diagnostics, USA) prior to testing.

### Block-based MgPa real-time PCR assay.

Clinical samples were extracted using the NucliSENS easyMAG (bioMérieux, France) and the MagNAPure 24 (Roche Diagnostics, Switzerland) systems according to the manufacturers’ instructions. For the easyMAG system, 1 ml of urine (off-board lysis) and 200 μl of swab samples (on-board lysis) were extracted using the serum protocol and eluted in 100 μl of NucliSENS elution buffer 3. For the MagNAPure 24 system, 1 ml of urine (Pathogen 1000 protocol) and 200 μl of swab samples (Pathogen 200 protocol) were extracted and eluted in 100 μl of the MagNAPure 24 elution buffer.

The block-based assay was a TaqMan probe-based real-time quantitative PCR assay that targets MgPa, the main adhesion protein of M. genitalium (16). This PCR was used for the detection and quantification of M. genitalium from the easyMAG and MagNAPure DNA extracts on the Applied Biosystems 7500 system (Applied Biosystems, USA) in a final reaction volume of 50 μl using 5 μl DNA template.

### Ethics.

All samples were anonymized and examined with the purpose for which they were originally submitted, and consequently, the study is considered quality assurance or quality development. As such, an ethics approval was not required according to Danish law no. 593 (14 June 2011) relating to ethics approval of research in health sciences.

## RESULTS

### Analytical sensitivity and inclusivity testing.

The analytical sensitivities for the RPMG Flex assay are presented in Table 4. The LOD values obtained were similar in urine and swab samples for all targets except for the M. genitalium A2059G 23S rRNA mutation type (M6593 strain), which had a slightly higher LOD in swab compared to urine samples. For all strains, the LOD was <400 copies/ml. For inclusivity testing, all additional M. genitalium strains were correctly detected in urine and swab matrices.

**TABLE 4:** Analytical sensitivity of the ResistancePlus MG Flexible assay for detection of M. genitalium and 23S rRNA mutation types and comparison with the MgPa assay for the detection of M. genitalium

M. genitalium 23S rRNA mutation type	Strain	LOD for:			
		RPMG Flex assay		MgPa assay (DNA extracted on the MP24 instrument)	
		Urine (genomes/ml)	Vaginal swab (genomes/ml)	Urine (genomes/ml)	Vaginal swab (genomes/ml)
WT	G37	157	157	339	1,321
A2058C	M6302	317	317	Not tested	Not tested
A2059G	M6593	147	220	Not tested	Not tested
A2058G	M6604	387	387	Not tested	Not tested
A2058T	M6926	151	151	Not tested	Not tested

The RPMG Flex assay demonstrated cross-reactivity to the M. genitalium A2059C 23S rRNA mutation type, which identified 90% of replicate samples when tested at 5,000 copies per sample.

For detection of the M. genitalium WT strain (G37) in urine samples, the sensitivity of the block-based MgPa PCR assay was 339 genomes/ml and 765 genomes/ml utilizing the MagNAPure 24 and NucliSENS easyMAG extraction platforms, respectively. For swab samples, the sensitivity for this assay was 1,321 genomes/ml and 1,338 genomes/ml, respectively, using each of the extraction platforms.

### Analytical specificity.

None of the 42 nontarget microorganisms gave a false-positive result, including closely related *Mycoplasma* species (see Table 2).

### Interfering substances.

None of the substances tested at concentrations that might be found in clinical specimens interfered with either the detection of the representative strain of M. genitalium or the indication of a false-positive result in the M. genitalium-negative specimens (Table 3). With urine specimens, assay interference may be observed in the presence of blood at a concentration greater than 0.4% vol/vol and bilirubin at a concentration greater than 0.18 mg/ml. With vaginal swab specimens, assay interference may be observed in the presence of Vagisil intimate powder at a concentration greater than 0.1% wt/vol.

### Reproducibility.

All reproducibility runs produced valid results with all samples correctly detected (see Tables S1 to S6 in the supplemental material). For all variables evaluated, analysis of variance components of the quantification cycle (Cq) values performed on positive panel members yielded overall coefficient of variation (CV) (%) ranges at or below 10% for the MgPa, 23S rRNA mutation, and internal control targets.

### Clinical specimens.

Among the 40 urine samples, 20 were positive (10 WT and 10 with MRMM) by primary reference testing, and 20 were negative. For M. genitalium detection in urine matrix, the RPMG Flex assay had 95% sensitivity and 100% specificity, while the block-based MgPa assay had 95% sensitivity and 100% specificity regardless of the extraction platform used (Table 5). All MRMM were correctly identified.

**TABLE 5:** Clinical sensitivity and specificity of the ResistancePlus MG FleXible assay (RPMG Flex)^*a*^

Assay	Matrix	Sensitivity (%)	% Correct assignment of MRMM	% Correct assignment of WT	Specificity (%)
RPMG Flex	Urine	95 (19/20)	100 (9/9)	100 (10/10)	100 (20/20)
MgPa-MagNAPure 24	Urine	95 (19/20)	NA	NA	100 (20/20)
MgPa-NucliSENS easyMAG	Urine	95 (19/20)	NA	NA	100 (20/20)
RPMG Flex	All genital swab samples (*n* = 122)	96 (55/57)	89 (26/29)	96 (25/26)	98 (64/65)
MgPa-MagNAPure 24	All genital swab samples (*n* = 122)	82 (47/57)	NA	NA	98 (64/65)
MgPa-NucliSENS easyMAG	All genital swab samples (*n* = 122)	84 (48/57)	NA	NA	98 (64/65)
RPMG Flex	Cervical swab (*n* = 63)	95 (35/37)	85 (17/20)	100 (15/15)	100 (26/26)
MgPa-MagNAPure 24	Cervical swab (*n* = 63)	76 (28/37)	NA	NA	100 (26/26)
MgPa-NucliSENS easyMAG	Cervical swab (*n* = 63)	78 (29/37)	NA	NA	100 (26/26)
RPMG Flex	Vaginal swab (*n* = 27)	100 (6/6)	2/2	4/4	95 (20/21)
MgPa-MagNAPure 24	Vaginal swab (*n* = 27)	100 (6/6)	NA	NA	95 (20/21)
MgPa-NucliSENS easyMAG	Vaginal swab (*n* = 27)	100 (6/6)	NA	NA	100 (21/21)
RPMG Flex	Female urethral swab (*n* = 11)	100 (4/4)	100 (2/2)	100 (2/2)	100 (7/7)
MgPa-MagNAPure 24	Female urethral swab (*n* = 11)	75 (3/4)	NA	NA	100 (7/7)
MgPa-NucliSENS easyMAG	Female urethral swab (*n* = 11)	75 (3/4)	NA	NA	100 (7/7)
RPMG Flex	Male urethral swab (*n* = 21)	100 (10/10)	100 (5/5)	100 (4/5)	100 (11/11)
MgPa-MagNAPure 24	Male urethral swab (*n* = 21)	100 (10/10)	NA	NA	100 (11/11)
MgPa-NucliSENS easyMAG	Male urethral swab (*n* = 21)	100 (10/10)	NA	NA	100 (11/11)

a NA, not applicable.

Among 137 genital swab specimens, 15 samples (11%) were excluded from the evaluation due to probe check failure (66%) or invalid internal control (33%). These failures were determined by the first version of the software; subsequent versions appear to be more tolerant. A total of 57 were positive (27 WT and 30 with MRMM). For M. genitalium detection in swab matrix, the RPMG Flex assay had 96.5% sensitivity (55/57) (95% confidence intervals [CI], 73 to 100%). MRMM was correctly identified in 26 (89.7%) of 29 evaluable samples with MRMM. However, five of these samples were negative in both of the DNA extracts by the block-based assay. For swab samples where both of the DNA extracts were positive in the block-based assay, 22 (95.7%) of 23 MRMM samples were correctly identified. For WT samples, 26 were evaluable, and 25 (96.2%) were correctly identified. For overall mutation detection, the RPMG Flex assay had a 93% (95% CI, 69 to 100%) positive agreement with sequencing for detection of MRMM.

Among the 65 swab negative samples, the RPMG Flex assay correctly identified 64, resulting in a 98.5% (95% CI, 75 to 100%) specificity. The block-based MgPa assay detected 47 of 57 positive genital swabs, for an 82.5% (95% CI, 61 to 100%) sensitivity (*P* < 0.0001 [McNemar test] compared to RPMG Flex) and had a 98.5% (95% CI, 75 to 100%) specificity with samples extracted on the MagNAPure 24; sensitivity and specificity were 84.2% (95% CI, 62 to 100%) (*P* < 0.0001 compared to RPMG Flex) and 98.5% (95% CI, 75 to 100%), respectively, when genital swab samples were extracted on the easyMAG system (Table 5).

## DISCUSSION

Global data for resistance to macrolides indicate an upward trend and thereby urge for timely and effective treatment of M. genitalium infection (17). As current methods for the diagnosis of M. genitalium and resistance-guided therapy involve specialist, multiassay, centralized diagnostic protocols (7), there exists a need for a rapid, decentralized, and comprehensive assay. Our data indicate that the RPMG Flex assay for the GeneXpert system could provide such a platform.

The qualitative real-time assay using the RPMG Flex cartridges for the GeneXpert system was sensitive, specific, and reproducible as documented by both retrospective data analyses and real-time head-to-head comparisons. When reagent preparation and sample setup were taken into consideration, we found the RPMG Flex assay easy to use compared to the MgPa assay. The MgPa assay for WT strains has previously documented an LOD value of 140 genomes/ml for urine and 3,500 genomes/ml for swab samples (16). By comparison, the RPMG Flex assay performed similarly for urine samples (157 genomes/ml) but markedly better for swab samples (LOD, 157 genomes/ml). Where strains with MRMM were concerned, the RPMG Flex assay showed marginally lower analytical sensitivity (Table 4). The significantly improved sensitivity for swab samples for the RPMG Flex assay is perhaps unsurprising given the input volume for swab samples in this assay (1 ml for the RPMG Flex assay, 200 μl for the easyMAG and MP24 platforms). This trend was also reproducible for the clinical swab specimens. Here again, the sensitivity differed significantly between the assays (96.5% for the RPMG Flex and 82.5% and 84.2% for the MP24 and easyMAG extracts, respectively, on the block-based MgPa assay [*P* < 0.0001]). Nevertheless, the RPMG Flex assay demonstrably provides for a more sensitive diagnostic tool than the current reference assay at this laboratory.

There was absolute agreement between the RPMG Flex and the block-based MgPa assays for both sample extraction methods in clinical urine specimens (sensitivity of 95%, specificity of 100%), vaginal swab specimens (sensitivity of 100%, specificity of 95%), and male urethral swab specimens (sensitivity of 100%, specificity of 100%; see Table 5). Overall, for clinical swab specimens, the RPMG Flex assay had enhanced sensitivity compared to the reference assay as discussed above. For all clinical samples tested, two urine samples had to be categorized as false negative, i.e., one was negative with MP24 extraction and RPMG Flex, whereas the other was false negative only in the easyMAG extraction. We decided to include these data in order to further illustrate the difficulty in obtaining concordance in samples with very low organism loads.

The clinical samples tested in this study were collected from 2009 to 2015 and had been stored at –20°C with a median storage time of 3.5 years. Furthermore, the total volume of sample available for each swab specimen was limited so that all swab specimens tested on all platforms were prediluted 3.5 times with sterile UTM. These factors coupled with the low input volume of the reference assay may likely have contributed to the inability to detect low-positive specimens by the block-based assay. The limit of detection, for both the RPMG Flex assay and the block-based assay, was further challenged in detecting low-load MRMM samples (Table 5). Notwithstanding and despite of the compromised nature of the clinical samples used in this study, the RPMG Flex assay demonstrated superior sensitivity. Lack of detection of MRMM in samples with low organism load is well described in the RPMG block-based assay (18) and is also a problem in several other commercially available combined assays detecting M. genitalium and MRMM in the same assay (19). This is most likely due to differences in the amplification efficacy of the MgPa detection target and the 23S rRNA gene MRMM target, where only probes for MRMM are present. Thus, the assay cannot distinguish between a WT M. genitalium strain and an MRMM-carrying strain with a false-negative 23S rRNA gene amplification. Therefore, for M. genitalium-positive 23S rRNA mutation not-detected samples, we would recommend an examination of Cq values for the MgPa target, and in cases where the value is high, laboratories should consider reporting these as positive for M. genitalium but indeterminate for MRMM when advising clinicians. Whether this approach can be used for the RPMG Flex assay was not evaluated in the present study. Furthermore, predilution of clinical specimens is otherwise not acceptable for diagnostic purposes, and the instructions for use (IFU) supplied by the manufacturer recommend 3-ml swab collection tubes as suitable for clinical specimens.

A total of 15 genital swab specimens reported either an error (10 of 15; probe check failure) or an invalid result (5 of 15; invalid Cq for the internal control) on the RPMG Flex assay on the ADF version available to this laboratory at the time of testing. Typically, these samples would be retested in standard diagnostic settings; however, due to the lack of additional sample material for repeat testing, we decided to exclude these samples from further analyses. For specimens that reported an invalid IC Cq, typically ≤16 cycles, we were still able to examine amplification profiles for the other two targets (M. genitalium and 23S rRNA) and determine M. genitalium test results for them. These results were not considered for final analysis and reporting. The IC Cq cutoff values have since been adjusted to 16 to 28 cycles by the manufacturer, which if available at the time of our testing, would have resulted in the inclusion of the five samples. Our investigation found that the ADF version 1 software was restrictive in its postrun analysis features, which may limit the use of this assay for research purposes. In addition, a locked ADF, where the end user is unable to adjust acceptance criteria for the IC Cq values, might result in wastage of valuable sample material, as was our experience. In contrast, a locked ADF is a need for CE IVD approval and provides much needed standardized acceptance criteria for M. genitalium results across laboratories that implement the RPMG Flex assay. In addition, the locked ADF would further lessen the need for specialized training for the interpretation and, consequently, reporting of diagnostic results.

The RPMG Flex assay showed no cross-reaction to other phylogenetically related or nontarget organisms (Table 2), including in the presence of Mycoplasma pneumoniae, the closest relative of M. genitalium, at 1 × 10^6^ genomes/ml (20). Confidence in the performance of the assay was further boosted when no interference to detection of M. genitalium or 23S rRNA mutations was evident in the presence of potentially interfering substances (Table 3).

When the RPMG Flex assay was assessed for variation in performance, all individual panel members were correctly identified for both positive and negative urine and swab samples. Preprepared panels were stored at –80°C for up to 2 weeks and thawed prior to testing, which did not appear to affect the sensitivity of the assay. The coefficient of variance was highest for the internal control target across the board (0.08 to 4.24% for urine samples; 0.53 to 2.63% for swab samples) but well within the acceptable range.

A strength of the present study is the thorough determination of the analytical performance as well as studies of interfering substances which may be encountered in clinical use. However, the relatively small number of clinical samples included in the evaluation as well as the need to dilute swab specimens to have sufficient volume to run all the assays is a limitation that needs to be taken into account.

The flexible cartridge program for the GeneXpert Dx system has facilitated the development of a simple, sensitive, and timely diagnostic assay for the simultaneous detection of M. genitalium and MRMM from clinical specimens. In addition, the RPMG Flex assay would provide for supplementary sexually transmitted infection (STI) testing at diagnostic laboratories across the world where the GeneXpert Dx CT/NG (Chlamydia trachomatis*/*Neisseria gonorrhoeae) assay is already in use. Our evaluation shows that the time taken for sample-to-answer for the RPMG Flex assay (2.55 h.) is somewhat shorter than the time taken by the block-based RPMG assay (3.25 h.) and with significantly less hands-on time (see Table S7 for a breakdown of our evaluation). Most importantly, the random access to testing made it feasible to run a single sample immediately after receipt in the laboratory, providing the ability to do near-patient testing without the need for batch testing.

The RPMG Flex assay described here simultaneously detects M. genitalium and MRMM. Our data indicate that the assay as it stands and notwithstanding further evaluation may prove to be a very useful and potent tool for resistance-guided therapy of M. genitalium.

## Supplementary Material

Supplemental file 1
