# Supplementary material for: Evaluation of the ResistancePlus MG FleXible Assay for Detection of Wild-Type and 23S rRNA-Mutated Mycoplasma genitalium Strains
Source: J Clin Microbiol. 2020 Feb 24;58(3):e01900-19. doi: 10.1128/JCM.01900-19 (PMC7041592; doi:10.1128/JCM.01900-19)
Supplement: Supplemental file 1 [file JCM.01900-19-s0001.pdf]

Supplementary data

Table S1: Urine samples: Summary of reproducibility data for the MgPa target

MG 23S rRNA type	n/N[a]	Agreement (%)	Mean Cq	Between-lot		Between-day		Between-operator		Between-run	
				SD	CV (%)	SD	CV (%)	SD	CV (%)	SD	CV (%)
A2058C	36/36	100	28.34	1.04	3.67	0.40	1.42	0.07	0.24	0.71	2.51
A2058G	36/36	100	28.24	1.23	4.37	0.65	2.30	0.05	0.17	0.69	2.44
A2058T	36/36	100	30.37	0.83	2.74	0.87	2.87	0.05	0.18	0.82	2.68
A2059G	36/36	100	29.42	1.18	4.03	0.90	3.05	0.13	0.45	0.95	3.23
WT	36/36	100	30.64	0.75	2.46	0.48	1.56	0.10	0.32	0.59	1.93
MG negative	36/36	100	--	--	--	--	--	--	--	--	--

[a] n/N = number of correctly identified samples/total number of samples tested

Cq = Quantification cycle

SD = standard deviation

CV = Coefficient of variation

Table S2: Urine samples: Summary of reproducibility data for the IC target

MG 23S rRNA type	n/N[a]	Agreement (%)	Mean Cq	Between-lot		Between-day		Between-operator		Between-run	
				SD	CV (%)	SD	CV (%)	SD	CV (%)	SD	CV (%)
A2058C	36/36	100	18.88	0.46	2.42	0.35	1.84	0.05	0.27	0.39	2.07
A2058G	36/36	100	18.75	0.50	2.66	0.33	1.77	0.11	0.59	0.36	1.90
A2058T	36/36	100	18.89	0.31	1.63	0.46	2.45	0.26	1.39	0.57	3.04
A2059G	36/36	100	18.61	0.68	3.67	0.13	0.71	0.02	0.08	0.28	1.49
WT	36/36	100	18.74	0.54	2.86	0.29	1.57	0.04	0.19	0.27	1.42
MG negative	36/36	100	20.42	0.87	4.24	0.47	2.36	0.04	0.18	0.58	2.90

[a] n/N = number of correctly identified samples/total number of samples tested

Cq = Quantification cycle

SD = standard deviation

CV = Coefficient of variation

Table S3: Urine samples: Summary of reproducibility data for the 23S rRNA mutation target

	n/N[a]	Agreement (%)	Mean Cq	Between-lot	Between-day	Between-operator	Between-run

MG 23S rRNA type				SD	CV (%)	SD	CV (%)	SD	CV (%)	SD	CV (%)
A2058C	36/36	100	28.34	0.29	1.12	0.38	1.46	0.08	0.31	0.43	1.64
A2058G	36/36	100	28.24	0.23	0.90	0.37	1.45	0.51	1.98	0.63	2.45
A2058T	36/36	100	30.37	0.58	2.15	0.30	1.11	0.89	3.28	0.80	2.96
A2059G	36/36	100	29.42	0.37	1.38	0.32	1.16	0.00	0.00	0.43	1.58
WT	36/36	100	--	--	--	--	--	--	--	--	--
MG negative	36/36	100	--	--	--	--	--	--	--	--	--

[a] n/N = number of correctly identified samples/total number of samples tested

Cq = Quantification cycle

SD = standard deviation

CV = Coefficient of variation

Table S4: Vaginal swab samples: Summary of reproducibility data for the MgPa target

	n/N[a]	Agreement (%)	Mean Cq	Between-lot	Between-day	Between-operator	Between-run

MG 23S rRNA type				SD	CV (%)	SD	CV (%)	SD	CV (%)	SD	CV (%)
A2058C	36/36	100	28.34	0.29	1.12	0.38	1.46	0.08	0.31	0.43	1.64
A2058G	36/36	100	28.24	0.23	0.90	0.37	1.45	0.51	1.98	0.63	2.45
A2058T	36/36	100	30.37	0.58	2.15	0.30	1.11	0.89	3.28	0.80	2.96
A2059G	36/36	100	29.42	0.37	1.38	0.32	1.16	0.00	0.00	0.43	1.58
WT	36/36	100	30.64	0.46	1.65	0.24	0.85	0.33	1.19	0.44	1.57
MG negative	36/36	100	--	--	--	--	--	--	--	--	--

[a] n/N = number of correctly identified samples/total number of samples tested

Cq = Quantification cycle

SD = standard deviation

CV = Coefficient of variation

Table S5: Vaginal swab samples: Summary of reproducibility data for the IC target

MG 23S rRNA type	n/N[a]	Agreement (%)	Mean Cq	Between-lot		Between-day		Between-operator		Between-run	
				SD	CV (%)	SD	CV (%)	SD	CV (%)	SD	CV (%)

A2058C	36/36	100	18.88	0.29	1.68	0.17	0.98	0.31	1.84	0.38	2.23
A2058G	36/36	100	18.75	0.47	2.63	0.16	0.92	0.42	2.37	0.38	2.14
A2058T	36/36	100	18.89	0.08	0.44	0.14	0.82	0.08	0.48	0.16	0.95
A2059G	36/36	100	18.61	0.24	1.35	0.18	1.05	0.09	0.53	0.25	1.43
WT	36/36	100	18.74	0.20	1.15	0.19	1.09	0.09	0.55	0.22	1.28
MG negative	36/36	100	20.42	0.37	2.17	0.20	1.12	0.52	2.97	0.45	2.60

[a] n/N = number of correctly identified samples/total number of samples tested

Cq = Quantification cycle

SD = standard deviation

CV = Coefficient of variation

Table S6: Vaginal swab samples: Summary of reproducibility data for the 23S rRNA mutation target

MG 23S rRNA type	n/N[a]	Agreement (%)	Mean Cq	Between-lot		Between-day		Between-operator		Between-run	
				SD	CV (%)	SD	CV (%)	SD	CV (%)	SD	CV (%)
A2058C	36/36	100	28.93	0.41	1.52	0.28	1.05	0.29	1.10	0.43	1.61
A2058G	36/36	100	32.99	0.56	1.85	0.37	1.23	0.13	0.43	0.65	2.13
A2058T	36/36	100	31.34	0.39	1.35	0.25	0.88	0.53	1.84	0.53	1.84

A2059G	36/36	100	30.60	0.91	3.30	0.62	2.27	0.57	2.07	0.88	3.21
WT	36/36	100	--	--	--	--	--	--	--	--	--
MG negative	36/36	100	--	--	--	--	--	--	--	--	--

[a] n/N = number of correctly identified samples/total number of samples tested

Cq = Quantification cycle

SD = standard deviation

CV = Coefficient of variation

Table S7: Workflow analysis for sample-to-answer for 16 samples with the RPMG Flex assay on the GeneXpert-16 system versus extraction on the easyMAG robot and plate-based MgPa PCR assay

16 Samples	MgPa[a] assay on the easyMAG and AB7500 instruments<br>Total Time: 3.25 hours (hh:mm)	RPMG[b] Flex Assay on the GeneXpert instrument<br>Total Time: 2.55 hours (hh:mm)
Office Work (Sample Registration/ Reporting)	00:30	00:30
Hands-on Laboratory Work		
DNA Extraction	00:10	00:00
Reaction Setup	00:20	00:20
Reaction Analyses	00:10	00:05
Hands-off Time		
Extraction Time	00:45	
Reaction Time	01:30	02:00

[a] – Detection of MG only

[b] – Detection of MG and MRMM
